# A chromosome-scale and haplotype-resolved genome assembly of carnation (*Dianthus caryophyllus*) based on high-fidelity sequencing

**DOI:** 10.3389/fpls.2023.1230836

**Published:** 2023-08-04

**Authors:** Heling Jiang, Xiaoni Zhang, Luhong Leng, Desheng Gong, Xiaohui Zhang, Junyang Liu, Dan Peng, Zhiqiang Wu, Yingxue Yang

**Affiliations:** ^1^ Center for Chinese Medicinal Omics and Floriculture, Kunpeng Institute of Modern Agriculture at Foshan, Foshan, China; ^2^ The Plant Genomics Research Center, Agricultural Genomics Institute at Shenzhen, Chinese Academy of Agricultural Sciences, Shenzhen, China

**Keywords:** *Dianthus caryophyllus*, genome assembly, genome annotation, chromosome synteny, phylogenetic analysis

## Abstract

*Dianthus caryophyllus* is an economic species often considered excellent cut flowers and is suitable for bouquets and gardens. Here, we assembled the haplotype-resolved genome of *D. caryophyllus* ‘Aili’ at the chromosome level for the first time. The total lengths of the two assembled haplotypes of carnation were 584.88 Mb for haplotype genome 1 (hap1) and 578.78 Mb for haplotype genome 2 (hap2), respectively. We predicted a total of 44,098 and 42,425 protein-coding genes, respectively. The remarkable structure variation was identified between two haplotypes. Moreover, we identified 403.80 Mb of transposable elements (TEs) in hap1, which accounted for 69.34% of the genome. In contrast, hap2 had 402.70 Mb of TEs, representing 69.61% of the genome. Long terminal repeats were the predominant transposable elements. Phylogenetic analysis showed that the species differentiation time between carnation and gypsophila was estimated to be ~54.43 MYA. The unique gene families of carnation genomes were identified in ‘Aili’ and previously published ‘Francesco’ and ‘Scarlet Queen’. The assembled and annotated haplotype-resolved *D. caryophyllus* genome not only promises to facilitate molecular biology studies but also contributes to genome-level evolutionary studies.

## 1 Introduction

Carnation (*Dianthus caryophyllus* L.) is a perennial herb of the Caryophyllaceae family (Tanase et al., 2012). It is native to the Mediterranean region and has been cultivated in Europe for more than 2,000 years (Arif et al., 2014). It is cultivated on a large scale in Germany, Hungary, Italy and the Netherlands. It is also cultivated in large quantities in countries such as Japan, Korea and Malaysia in Asia (Chandra et al., 2016). It is one of the most widely used flowers in the world. More than 300 *Dianthus* species were produced worldwide until now (Yagi et al., 2013). Carnations include many varieties and hybrids, with abundant flowers in many shapes of single, half and double (Wang et al., 2020). Carnations have colors in crimson, blush, purple, red, scarlet, white and tan. There are also striped, dotted, spotted and veined carnations with smooth or slightly pleated petal edges (Smith et al., 1992). According to flower type, it is divided into Large Flowered Carnations such as Sims and Mediterranean, Mini Carnations such as Rony and Exquisite, Small Carnations such as Dianthini. They bloom almost continuously in the greenhouse and are suitable for bouquets and gardens. Carnations are excellent cut flowers (Jose et al., 2017). Dwarf varieties can also be used for potted ornamental. They are valued for their beauty, charm, clove-like fragrance and long-lasting freshness.

The previously released carnation genome of ‘Francesco’ and ‘Scarlet Queen’ provides a foundation for comparative and genomics analyses of carnations (Yagi et al., 2013; Zhang et al., 2022). Most carnation cultivars are diploid species (2n = 30) with a small genome approximately 550-630 Mb (Jose et al., 2017), which is about a fourth that of *Arabidopsis* (Agulló-Antón et al., 2013). Heterozygosity can ensure the diversity of the population. Heterozygotes are usually more stable in a mutated environment. Heterozygous genotypes can have higher relative fitness than homozygous dominant genotypes or homozygous recessive genotypes (Oostermeijer et al., 1995). This is called heterozygous advantage. Carnation is highly heterozygous (Yagi, 2015). For species with high heterozygosity, differences between the homologous chromosomes and their hidden genetic information cannot and should not be ignored (Cheng et al., 2022). However, for species with low heterozygosity (e.g., heterozygosity less than 0.05%), the differences between the two chromosomes are typically ignored during genome assembly, and a reference genome is constructed to represent the species. Compared to species with low heterozygosity and a lower abundance of repetitive sequences, highly heterozygous species (such as those with perennial self-incompatibility or distant hybridization) present greater challenges in haplotype genome assembly. The assembly of genomes from highly heterozygous species is inherently difficult (Pryszcz and Gabaldón, 2016).

In this study, the widely cultured cultivar, ‘Aili’, was selected for genome sequencing. The chromosome-scale and haplotype-resolved genome assembly of *D. caryophyllus* was presented. The genome was sequenced by a combination of Pacific Biosciences (PacBio) high-accuracy long-read (HiFi) genome sequencing and high-throughput chromosome conformation capture (Hi-C) technologies. In total, we assembled and anchored 36.9 Gb of HiFi reads to 15 chromosomes. Two assembled haplotypes of carnation were 584.88 Mb for haplotype genome 1 (hap1) and 578.78 Mb for haplotype genome 2 (hap2), respectively. We obtained scaffold N50 lengths of 19.84 Mb for hap1 and 25.17 Mb for hap2, respectively. High Benchmarking Universal Single-Copy Orthologs (BUSCO) completeness rates (97.50% for hap1 and 97.40% for hap2) confirmed the high quality of the genome assembly. There were remarkable structure variations between two haplotypes. Phylogenetic analysis was performed and the carnation genome was shown to undergo a whole genome triplication (WGT) event. This haplotype-resolved genome assembly provide valuable resources for carnation improvement and comparative genomics research.

## 2 Materials and methods

### 2.1 Plant materials and genome sequencing

Carnation variety ‘Aili’ (phenotype shown in the **Supplementary Figure 1**) was grown in the experimental field of the Comprehensive Experimental Base of Shenzhen Institute of Agricultural Genomics, Chinese Academy of Agricultural Sciences (located at 22°601231N, 114°500634E), Shenzhen, Guangdong Province, China. Young leaves of ‘Aili’ were collected and genomic DNA was prepared by the cetyltrimethylammonium bromide (CTAB) method. After obtaining high quality purified genomic DNA samples, we constructed a 15kb insert size PCR-free SMRT library and sequenced it using the PacBio Sequel II platform, which gave us 36.90 Gb (60× coverage) data. Meanwhile, we used the BGI sequencing platform to construct a library of DNA fragments with an insert size of approximately 150 bp and then sequenced using sequencing technology to generate a total of 16.57 Gb (27× coverage) of raw reads. Raw sequencing data was evaluated for quality using FastQC (v0.11.9) (Brown et al., 2017). To anchor contigs onto the chromosome, genomic DNA was extracted for the Hi-C library from ‘Aili’. We then passed the constructed Hi-C library through the MGl-2000 platform to obtain 114× of 70.65 Gb Hi-C data and after quality control analysis with fastp (0.23.2) (Chen et al., 2018) and removal of linker sequences and low-quality sequences, 70.28 Gb of Hi-C clean reads were obtained. The stem, leaf, and flower tissues of ‘Aili’ were extracted, and RNA library was prepared for transcriptome sequencing. We constructed a paired-end library with an insert size of 150 bp and sequenced it using the Illumina Novaseq platform, generating a total of 6.52 Gb of paired-end reads.

### 2.2 Genome sequences assembly and quality evaluation

Before *de novo* assembly of the carnation genome, we utilized high-quality BGI paired-end reads to estimate genome size and heterozygosity rates using Genomescope (1.0.0) (Vurture et al., 2017) software with *k*-mer counts calculated from Jellyfish (2.3.0, kmer=21, histogram=50000) (Marcais and Kingsford, 2011). We used hifiasm (0.16.1) (Cheng et al., 2021) software for Hifi read-based assembly assisted by Hi-C data (both Hifi reads and Hi-C reads were input to hifiasm), and two haplotype-resolved contig sets were obtained. These contigs were evaluated using the *k*-mer analysis tool KAT (V2.4.2, comp mode) (Mapleson et al., 2017). We used the blastn (2.110) software to remove highly similar sequences in both the chloroplast and mitochondrial genomes from two contigs sets. Sequences that satisfy both the similarity of more than 95% and the length of less than 1Mb were filtered for deletion.

Next, we performed chromosome-level scaffolding using Hi-C data. First, we further checked the reliability of Hi-C data with HiC-Pro (3.1.0) (Servant et al., 2015) using the alignments of Hi-C paired-end reads to the assembled contigs from Bowtie2 (3.1.0) (Langmead and Salzberg, 2012). Then the scaffolding was performed for each of the two sets of haplotype-resolved contigs using 3D-DNA (version 180114, with parameter -r 0) (Dudchenko et al., 2017) with preprocessed Hi-C data from Juicer (1.6, -s DpnII) (Durand N.C. et al., 2016). After generating chromosome-level assembly with automatic tools, we did manual curation according to the Hi-C heatmap visualized by juicebox (1.11.08) (Durand N. et al., 2016) to further improve the quality. More specifically, once unexpected strong signals appear far away from the diagonal in the Hi-C heatmap, we change the order of the related contigs until they disappear. To evaluate the correctness of the assembly, the ‘72L’genetic maps of carnation previously published (Yagi et al., 2016) were used, and the mapping was carried out using ALLMAPS (Tang et al., 2015) software. QUAST (5.0.2) (Mikheenko et al., 2018), Merqury (1.3, count k=21) (Rhie et al., 2020) and BUSCO (v5, embryophyta_odb10) (Simao et al., 2015) were also run for assessments of genome assembly quality. Additionally, the BGI and Hifi reads were mapped back to the assembly, and the homozygous single nucleotide variation rate was calculated from the results of GATK (4.0.5.1, –filter-expression “(QD <2.0) || (FS >60.0) || (MQRankSum < -12.5) || (ReadPosRankSum < -8.0)” –filter-name “PASS”) (Van der Auwera et al., 2013) to evaluate the accuracy of the assembly. Finally, we also used long terminal repeat (LTR) assembly index (LAI) calculated by LTR_Finder (v2.9.0, -u 4.02e-9) (Xu and Wang, 2007) to evaluate genome quality.

### 2.3 Genome annotation and synteny analysis

Before annotating the encoded protein in the genome, we first annotated original TEs based on The Extensive *de novo* TE Annotator (EDTA) (v1.9.4, –anno 1 –force 1 –debug 1 -sensitive 1) (Ou et al., 2019) for generating high-quality non-redundant TE libraries for genome-wide TE annotation, including long terminal repeat retrotransposons (LTR-RTs), DNA with terminal inverted repeat (TIR) sequences transposons and other repetitive sequences, etc. Using exonerate (2.2.0, –showalignment false –showtargetgff true –percent 50 –bestn 1 –minintron 10 –maxintron 100000) (https://github.com/nathanweeks/exonerate) and Augustus (3.4.0) (Stanke et al., 2004) for gene prediction based on homologous proteins, selected genomes were from *Arabidopsis thaliana* (Sloan et al., 2018), *Beta vulgaris* (Dohm et al., 2014), *Carica papaya(* Ming et al., 2008 *)*, *D. caryophyllus* ‘Scarlet Queen’ (Zhang et al., 2022), *Oryza sativa* (Jain et al., 2019), *Rosa chinensis* (Raymond et al., 2018), *Solanum lycopersicum* (Takei et al., 2021), and *Vitis vinifera* (Jaillon et al., 2007). For transcript-based predictions, we used Trinity (v2.2.0, –genome_guided_max_intron 10000) (Haas et al., 2013), HISAT2 (2.2.1) (Kim et al., 2015) to perform RNA-Seq first Transcript assembly for generating coding regions, and then used TransDecoder (v5.5.0) (https://github.com/TransDecoder/TransDecoder) to identify candidate coding regions in transcript sequences (TransDecoder identifies candidate coding regions within transcript sequences those generated by *de novo* RNA-Seq transcript assembly using Trinity and HISAT2), and the PASA software was used to predict gene structure by aligning the cDNA sequence to the genomic sequence. Augustus, SNAP (https://github.com/KorfLab/SNAP) and GlimmerHMM (Majoros et al., 2004) were used for *de novo* gene prediction. In summary, the multiple gene sets predicted by the above software were integrated into a more complete gene set using evince modeler (EVM) (1.1.1) (Haas et al., 2008). Collinearity analysis of two haplotype genomes was performed by MUMmer (4.0.0beta2, –filter -i 90 -l 10000) (Marçais et al., 2018) and SyRI (1.5.4, -k -F B) (Goel et al., 2019). The intragenome synteny blocks were determined by JCVI (v1.2.7) (Tang et al., 2008) and MCScanX (Wang et al., 2012).

After the genome structure annotation is completed, we first searched the InterPro database through InterProScan (5.21, -goterms -iprlookup -pa -f TSV -dp) (Jones et al., 2014) to annotate the protein structure domain, so as to obtain the Gene Ontology (GO) (Consortium, 2004) regulatory pathway corresponding to each gene, and then through the three databases of SwissProt (Boeckmann et al., 2003), Non-Redundant Protein Sequence Database (NR) (Sayers et al., 2020) and eukaryotic orthologous groups (KOG) (Koonin et al., 2004) BLASTp (evalue 1e-5 cut off) alignment for functional annotation of protein-coding genes. The process of annotating non-coding RNA involved identification of microRNAs (miRNAs), ribosomal RNAs (rRNAs), and transfer RNAs (tRNAs). To accomplish this, Rfam (Griffiths-Jones et al., 2005) and Infernal software (1.1.4, –cut_ga –rfam –nohmmonly –fmt 2) (Nawrocki and Eddy, 2013) were employed to identify various categories of non-coding RNA.

### 2.4 Comparison among the three carnation genomes

To investigate the differences among the single haplotype carnation genome assembled in this study and two previously published non-haplotype carnation genomes ‘Francesco’ and ‘Scarlet Queen’, we annotated the protein sequence of ‘Aili’ (selecting hap2 for comparison), and downloaded protein sequences of ‘Francesco’ and ‘Scarlet Queen’ to identify orthologous gene clusters. The comparison results were demonstrated using the online tool OrthoVenn2 (https://orthovenn2.bioinfotoolkits.net/). GO enrichment analyses were performed on specific genes using EggNOG-mapper (http://eggnog-mapper.embl.de/) and TBtools (Chen et al., 2020). Hiplot Pro (https://hiplot.com.cn/) was used to visualize final files. Kyoto Encyclopedia of Genes and Genomes (KEGG) (Kanehisa and Goto, 2000) analysis was performed by GFAP (Xu et al., 2022).

### 2.5 Phylogenetic analysis and estimation of divergence times

Besides the annotated proteins of carnation, protein annotations from ten species, including *A. thaliana*, *B. vulgaris*, *Gypsophila paniculate* (Li et al., 2022), *Haloxylon ammodendron* (Wang et al., 2022), *O. sativa*, *Portulaca amilis* (Gilman et al., 2022), *Selenicereus undatus* (Chen et al., 2021), *Spinacia oleracea* (Cai et al., 2021) and *D. caryophyllus* ‘Francesco’ and ‘Scarlet Queen’ were utilized to explore the evolutionary positioning of carnation. Gene family information was obtained by clustering with the OrthoFinder (v2.5.4, -S diamond -M msa) (Emms and Kelly, 2019). The MCMCTREE package implemented in PAML (4.9) (Yang, 2007) was used to estimate the divergence time between species. We used divergence times obtained from the TimeTree database (http://www.timetree.org/) to calibrate our model, including *H. ammodendron* and *S. oleracea* (23.4–66.0 MYA), *P. amilis* and *S. undatus* (11.9–32.1 MYA), *B. vulgaris* and *S. oleracea* (23.6–62.4 MYA), *D. caryophyllus* and *P. amilis* (53.4–78.9 MYA).

### 2.6 Gene family expansion and contraction

A contraction-expansion analysis was performed on the phylogenetic tree using CAFE (v5, -p 0.05 -r 10000) (De Bie et al., 2006) combined with gene family clustering results and species divergence times. We also conducted GO enrichment analysis (p < 0.05) for significantly contracted and expanded gene families to each type of carnation genome, using the same method above.

### 2.7 Whole-genome duplication event identification

In order to investigate the genome duplication events that occurred in carnation, we used beet as the reference and compared the protein sequences using blastp (2.11.0), the parameter is “-evalue 1e-5 -max_target_seqs 5”, then collinear gene pairs were screened out by MCScanX, and JCVI (–minspan=30) were used to conduct in-depth auxiliary analysis. To further validate the occurrence of paleopolyploidization events, we computed the synonymous substitution rates (Ks) for every pair of collinear genes in the genomes of *Lactuca sativa*, *D. caryophyllus* ‘Aili’, and ‘Scarlet Queen’, and subsequently employed ggplot2 to create a graphical representation of the results.

## 3 Results

### 3.1 Haplotype-resolved genome assembly

We sequenced the carnation diploid genome and obtained a total of 36.9 Gb PacBio long reads (**Supplementary Table 1** and **Supplementary Figure 2**). The genome size was estimated to be 581.70 Mb using *k*-mer analysis, with 1.23% heterozygosity, and a duplicate rate of 0.45% (**Supplementary Figure 3**). We assembled two haplotype genomes at the contigs level. The total lengths of the original contigs were 584.88 Mb for hap1 and 578.78 Mb for hap2, respectively. *k*-mer comparison plots for the two haplotype contigs indicated the correct assembly (**Supplementary Figure 4**). After filtering out the mitochondria and chloroplasts related contigs, the generated hap1 included 245 contigs with a N50 of 19.84 Mb, hap2 includes 189 contigs with a N50 of 25.17 Mb (**Table 1**).

**Table 1:** Statistic result of the two haplotype assemblies of the *D. caryophyllus* genome.

Type	Aili hap1	Aili hap2
Raw contigs	321	195
Total raw length	584,881,303	578,777,982
Cleaned contigs	245	189
Total cleaned length	582,387,105	578,488,033
Largest contig	41,281,361	40,164,664
GC (%)	38	38
N50	19,844,739	25,168,369
N75	12,037,430	13,528,445
L50	11	9
L75	21	16
N’s per 100 kbp	0	0
Length of Chr01	43,232,911	43,471,602
Length of Chr02	41,126,408	40,317,290
Length of Chr03	44,164,944	40,810,358
Length of Chr04	41,281,361	40,164,664
Length of Chr05	37,740,359	37,957,961
Length of Chr06	36,444,141	35,831,868
Length of Chr07	37,464,026	36,944,158
Length of Chr08	35,758,108	37,806,305
Length of Chr09	36,222,050	37,926,771
Length of Chr10	35,169,524	35,478,709
Length of Chr11	38,022,789	37,063,354
Length of Chr12	33,869,868	34,209,210
Length of Chr13	37,300,756	37,238,286
Length of Chr14	35,703,483	36,186,748
Length of Chr15	33,995,030	34,885,108
Total length of anchored chromosome	567,495,758	566,292,392

**Figure 1:**
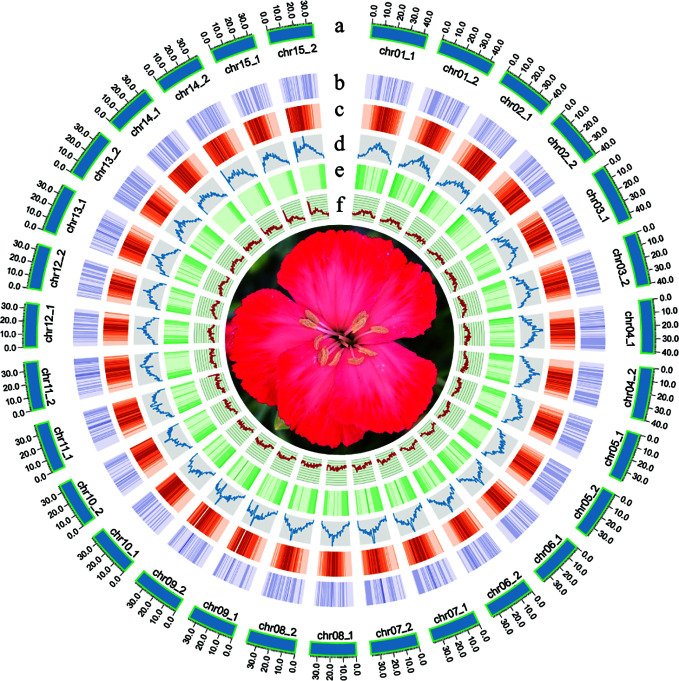
Flower traits of *D. caryophyllus* ‘Aili’ and Circos plot of the *D. caryophyllus* genomic features between the two haplotype assemblies. **(A)** chromosome length in Mb **(B)** the density of all Copia-Long terminal repeats (LTRs) **(C)** the density of all Gypsy-LTRs **(D)** the density of all LTRs **(E)** gene density **(F)** GC content in 500 kb windows.

The quality of Hi-C paired-end reads were validated by mapping to the assembled contigs (**Supplementary Table 2**). Two chromosome-level haplotype-resolved assembly of the diploid carnation were successfully obtained after Hi-C scaffolding (**Figure 1**). The total base numbers of chromosomes anchored to each haplotype genome were 567.50 Mb and 566.29 Mb, respectively. Chromosome length of hap1 ranged from the shortest 33.87M to the longest 43.23M, and the length of the hap2 chromosome ranged from the shortest 34.21M to the longest 43.47M. The Hi-C heat map indicated the Hi-C interaction signal was strong and the chromosome size was consistent, verifying the successful chromosome assembly (**Supplementary Figure 5**). The statistical GC content for hap1 and hap2 were 37.55% and 37.60%, respectively (**Supplementary Figure 6**). By conducting collinearity analysis with the genetic map of carnation ‘72L’, we found that both newly assembled haplotype genomes showed higher collinearity with ‘72L’ (**Supplementary Figures 7, 8**). We remapped BGI reads and HiFi reads to two assembled haplotype genomes and the mapping rates were statistically analyzed (**Supplementary Table 3**). Additionally, by calculating the single-base accuracy, we obtained a 99.99% assembly accuracy for both haplotype genomes (**Supplementary Table 4**).

Completeness and accuracy of genome assemblies were assessed using BUSCO. High BUSCO completeness rates (97.50% for hap1 and 97.40% for hap2) confirmed the high quality of the genome assembly (**Supplementary Figure 9**). Meanwhile, the statistical results of the two previously published genomes of *D. caryophyllus* are shown in **Supplementary Table 5**. The BUSCO completeness rates were reported as 97.15% for ‘Scarlet Queen’ and 97.00% for ‘Francesco’, both of which were lower than the completeness rates of the two diploid genomes assembled in this study. The LAI were 22.13 for hap1 and 22.20 for hap2, respectively (**Supplementary Figure 10**). Based on *k*-mer analysis, we calculated a consensus quality value (QV) of 59.96 and a *k*-mer completeness of 83.57% for hap1, and a consensus QV of 60.05 and a *k*-mer completeness of 83.40% for hap2 (**Supplementary Table 6**). These results provided evidence that we had obtained a high-quality carnation genome. At the same time, we compared the synteny of these two haplotype genomes with the published carnation genome, and found that the two carnation genome sequences are highly consistent (**Supplementary Figures 11, 12**). The above results indicated that both carnation haplotype genomes were assembled with high accuracy and continuity.

### 3.2 Differences between haplotypes

**Figure 2:**
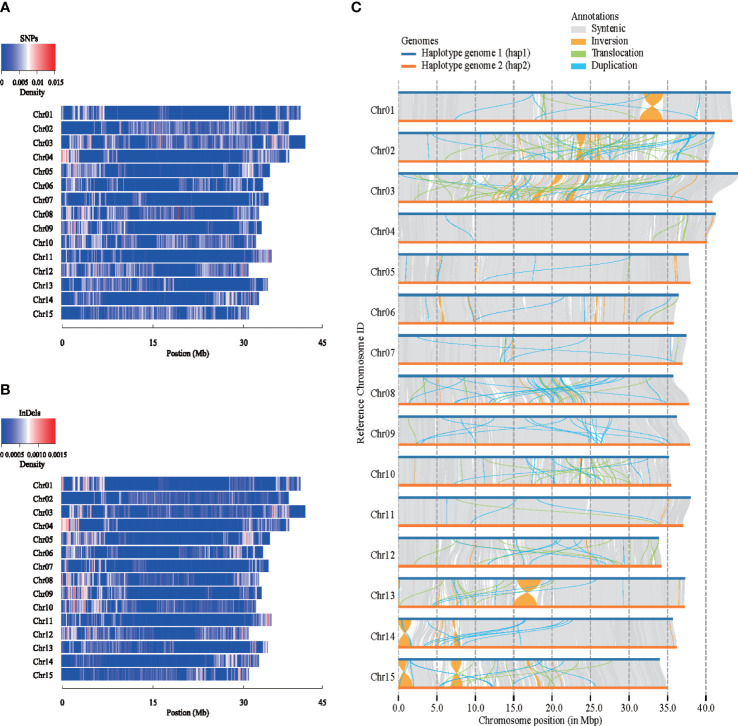
Structure variations between two carnation haplotype genomes. **(A)** Density distribution of single nucleotide polymorphisms (SNPs) among different chromosomes. **(B)** Density distribution of insertions or deletions of DNA segments (InDels) among different chromosomes. **(C)** Collinearity comparison between two haplotypes.

Structure variation analysis was performed on the two carnation haplotype genomes assembled for the first time (**Figure 2**), the map of single nucleotide polymorphisms (SNPs) and insertions or deletions of DNA segments (InDels) density distribution on 15 chromosomes was obtained, taking hap1 as reference (**Figures 2A, B**). From the perspective of the variation trend, the SNPs and the InDels density map were basically consistent. There were many variations at both ends of the Chr01, Chr03, Chr04, Chr06, Chr07, Chr08, and Chr09 chromosomes. Structure variations were found between hap1 and hap2 (**Figure 2C**). We detected a total of 27,299 SVs between haplotype genomes, of which duplications (DUPs, 10,087), ranging in size from 199 to 115,527 bp, and translocations (TRANSs, 3,970), ranging in size from 199 to 63,511 bp. The other SVs were 13,242 inversions (INVs), which ranged in size from 204 to 3,018,929 bp. Syntenic analysis of a genomic variation region was shown in **Supplementary Figure 13A**. In the syntenic block, one gene variation between hap1 and hap2 was taken as an example (**Supplementary Figure 13B**).

### 3.3 Genome annotation

In the carnation genome of ‘Aili’, the transposable elements sequences accounted for 69.34% (hap1) and 69.61 (hap2) (**Table 2**), respectively, which were basically consistent with the published genome of ‘Scarlet Queen’, which accounted for 70.62% (**Supplementary Table 7**) of the transposable element sequences. Transposable elements sequences were summarized in **Table 2**, among which the LTR retrotransposons account for 52.80% of the genome in hap1, of which 9.23% are copia type, and the remaining 25.29% are Gypsy type; LTR account for 53.46% of the genome in hap2, of which 8.95% were copia type, and the remaining 26.20% were Gypsy type. In hap1, the terminal inverted repeat (TIR) accounted for 8.32% of the total genome size, nonLTR accounted for 0.08%, and nonTIR accounted for 8.14%; in hap2, TIR sequences accounted for 8.57% of the total genome size, nonLTR accounted for 0.10%, and nonTIR accounted for 7.48%.

**Table 2:** Repetitive sequence classification statistics.

Type of transposable elements (TEs)		Aili hap1			Aili hap2		
		Number	Length (bp)	Percent (%)	Number	Length (bp)	Percent (%)
long terminal repeat (LTR)	Copia	50,193	53,740,552	9.23	50,650	51,800,010	8.95
	Gypsy	112,115	147,270,203	25.29	125,323	151,538,941	26.20
	unknown	139,570	106,487,071	18.28	138,514	105,894,456	18.31
terminal inverted repeat (TIR)	CACTA	15,876	6,132,746	1.05	18,185	7,160,947	1.24
	Mutator	39,015	12,738,939	2.19	35,311	11,747,003	2.03
	PIF_Harbinger	9,686	2,886,856	0.50	10,064	3,088,590	0.53
	Tc1_Mariner	72,892	17,679,196	3.04	78,139	18,985,346	3.28
	hAT	26,032	8,985,266	1.54	26,089	8,597,080	1.49
nonLTR	LINE_element	979	456,782	0.08	978	605,450	0.10
nonTIR	helitron	82,380	25,656,564	4.41	77,108	23,613,035	4.08
	repeat_region	49,931	21,702,523	3.73	44,907	19,667,438	3.40
total		598,743	403,799,656	69.34	605,268	402,698,296	69.61

Through EVM integration, we predicted a total of 44,098 and 42,425 protein-coding genes (**Figure 1** and **Supplementary Table 8**), and the BUSCO values were 97.00% and 97.10% for hap1 and hap2, respectively (**Supplementary Table 9**), confirming the high quality of our annotation. Combined with the predicted number of 43,266 genes in the published carnation genome of ‘Scarlet Queen’, the number of genes we predicted for the two haplotypes were basically consistent with the comparable number of genes in the carnation species. Finally, 91.59% and 91.34% of predicted genes in the two haplotype genomes were functionally annotated using different databases, respectively (**Supplementary Table 10**). The comparison of the number of genes in ‘Aili’ two haplotypes and ‘Scarlet Queen’ were shown in **Supplementary Figure 14**. Regarding the prediction of non-coding RNA, we identified 1,509 tRNAs, 91 miRNAs, 4,972 rRNAs, and 2,283 small nuclear RNA (snRNA) in hap1, however, 1,426 tRNAs, 91 miRNAs, 4,894 rRNAs, and 2,318 snRNAs were predicted in hap2 (**Supplementary Table 11**).

### 3.4 Comparison among the three carnation genomes

**Figure 3:**
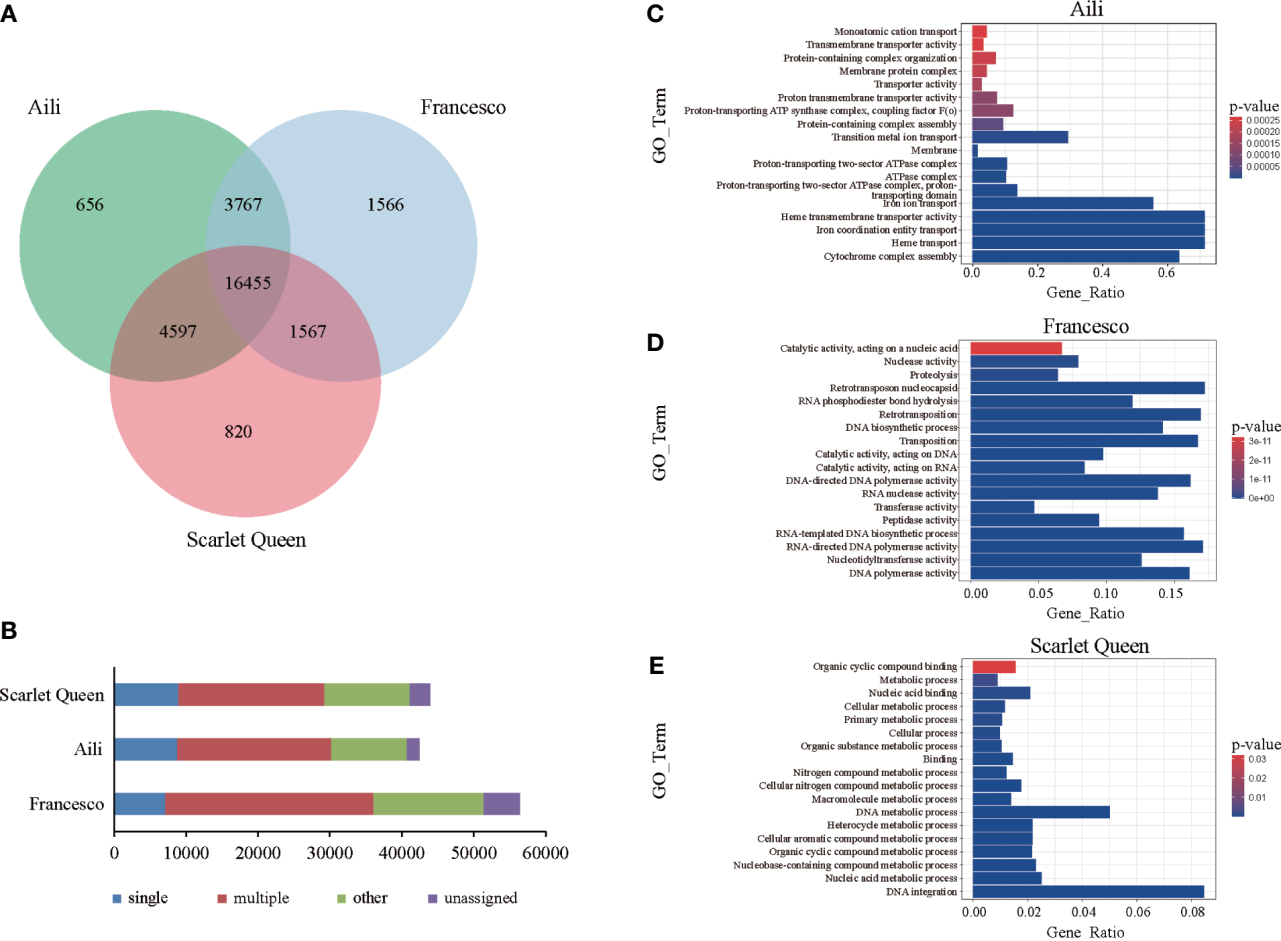
Comparison between the three carnation genomes. **(A)** The Venn diagram shared and unique gene families were compared between ‘Aili’ and two other *D. caryophyllus* genomes. **(B)** Genes in different groups of three **(*D*)** *caryophyllus* genomes were shown and the values are shown with bar charts. **(C-E)** Venn diagrams showing Gene Ontology (GO) annotation of the unique set of genes in carnation ‘Aili’, ‘Francesco’ and ‘Scarlet Queen’, respectively.

In order to explore the differences among the assembled *D. caryophyllus* ‘Aili’ genome and the previously published carnation genomes of ‘Francesco’ and ‘Scarlet Queen’, the gene family cluster analysis was performed in which hap2 of ‘Aili’ was chosen for comparison (**Figure 3A**). General statistics are presented as a Venn diagram, the result showed that there were 16,455 gene families common in the three carnations, and 656 gene families were unique to ‘Aili’.

We further compared the distribution of gene numbers in these clustered gene families. ‘Aili’ genes were classified as 4,865 single-copy, 8,525 multiple-copy, 27,320 others, and 1,715 unassigned genes (**Figure 3B** and **Supplementary Table 12**). ‘Francesco’ genes were classified as 3,751 single-copy, 12,550 multiple-copy, 35,103 others, and 4,978 unassigned genes. ‘Scarlet Queen’ genes were classified as 4,867 single-copy, 8,309 multiple-copy, 27,956 others, and 2,793 unassigned genes. GO annotation was performed on the unique gene families of the three carnations (**Figures 3C–E**). The results showed that ‘Aili’-specific genes were mainly enriched in heme transmembrane transporter activity, iron coordination entity transport, heme transport and cytochrome complex assembly (**Figure 3C** and **Supplementary Table 13**). ‘Francesco’-specific genes were mainly enriched in RNA-directed DNA polymerase activity, nucleotidyltransferase activity and DNA polymerase activity. (**Figure 3D** and **Supplementary Table 14**). ‘Scarlet Queen’-specific genes were mainly enriched in nucleic acid metabolic process and DNA integration (**Figure 3E** and **Supplementary Table 15**). The GO analysis showed that gene function in ‘Aili’ differed from that of ‘Francesco’ or ‘Scarlet Queen’. The enriched cytochrome complex assembly in ‘Aili’-specific genes may explain the color difference of three varieties, as cytochromes are related to the color trait. KEGG enrichment results also showed functional differences between three unique gene families (**Supplementary Figure 15**).

### 3.5 Evolutionary analysis of *D. caryophyllus* ‘Aili’

**Figure 4:**
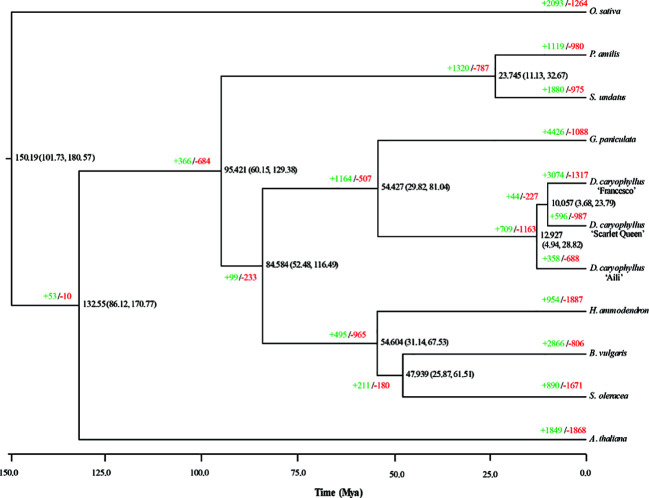
Phylogenetic tree and expanded and contracted gene families for *D. caryophyllus* and ten otherspecies. The numbers of expanded (in green) and contracted gene families (in red) are shown at the nodes in the phylogenetic tree. The divergence time is given in millions of years.

To investigate *D. caryophyllus* ‘Aili’ genome evolution, we compared its genome to those of other plant species in Caryophyllales, taking *A. thaliana* and *O. sativa* as two outgroups (**Figure 4**). A phylogenetic tree constructed from 430 single-copy orthologs indicated the phylogenetic relationships of nine genomes (including the three carnation genomes ‘Aili’, ‘Francesco’ and ‘Scarlet Queen’) from Caryophyllaceae, Amaranthaceae, Portulacaceae, and Cactaceae. The divergence time estimation revealed that Caryophyllaceae and Portulacaceae diverged about 84.58 million years ago, and Caryophyllaceae and Cactaceae diverged about 95.42 million years ago. In addition, the divergence time between the two genera *G. paniculata* and *D. caryophyllus* under the order Caryophyllales was about 54.43 million years ago. Notably, *D. caryophyllus* ‘Aili’ and ‘Scarlet Queen’ had more contracted gene families (688 for ‘Aili’ and 987 for ‘Scarlet Queen’) than expanded ones (358 for ‘Aili’ and 596 for ‘Scarlet Queen’). In contrast, *D. caryophyllus* ‘Francesco’ had more expanded gene families (3,074) than contracted ones (1,317) (**Figure 4**). The Gene Ontology (GO) enrichment terms of the expanded gene families for *D. caryophyllus* and *G. paniculata* were shown in **Supplementary Figure 16**. It showed that genes among expanded families of genes in *D. caryophyllus* ‘Aili’, ‘Francesco’ and ‘Scarlet Queen’ were preferentially enriched in trehalose metabolism in response to stress, alpha-amylase activity and response to auxin, respectively.The most enriched genes in expanded families in *G. paniculata* was inner mitochondrial membrane protein complex. The GO enrichment terms of the contracted gene families for *D. caryophyllus* were shown in **Supplementary Figure 17**. It showed that genes in contracted families in *D. caryophyllus* ‘Aili’, ‘Francesco’ and ‘Scarlet Queen’ were preferentially enriched in telomere organization, regulation of vegetative meristem growth and response to auxin, respectively.

### 3.6 Whole genome triplication

**Figure 5:**
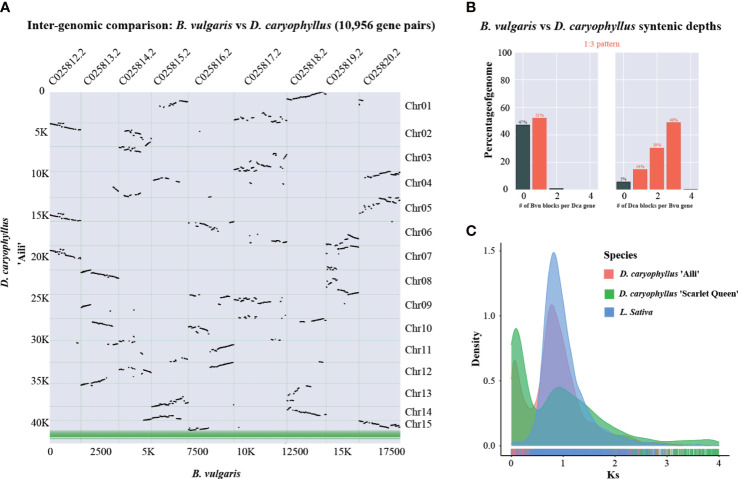
Whole-genome triplication event identification. **(A)** Syntenic dot plot illustrating the comparative analysis of the *D. caryophyllus* and *B. vulgaris* genomes. Chr01-Chr15 are 15 chromosome-scale pseudomolecules of the *D. caryophyllus*. **(B)** Syntenic depth ratio between the *D. caryophyllus* and *B. vulgaris* **(C)** Distribution of the synonymous substitution (Ks) rates for *D. caryophyllus* ‘Aili’ and ‘Scarlet Queen’ and *L. sativa* genome which experienced a recent WGT event.

For analyzing whole genome duplication events, *B. vulgaris* could serve as a reference species since it did not experience recent polyploidy event (Xu et al., 2017). The syntenic dot plot analysis indicated that there were three *D. caryophyllus* blocks in each *B. vulgaris* genome region (**Figure 5A**). And Syntenic depth ratio indicated a 1:3 pattern between *B. vulgaris* and *D. caryophyllus* (**Figure 5B**). Thus, it provided evidence for a WGT event in *D. caryophyllus*. Moreover, frequency distribution of synonymous substitution rates (Ks) for *D. caryophyllus* and *L. sativa* which experienced a recent WGT event (Badouin et al., 2017), supported the occurrence of whole genome polyploidy event (**Figure 5C**).

## 4 Discussion

Rapid advances in the development of genome sequencing in recent years has offered useful tools to acquire high-fidelity genomes to uncover new and potentially unexpected biological findings. However (Pervez et al., 2022; Sun et al., 2022), the genome information of many ornamental plant species is not known (Zheng et al., 2021), which hampers the research on plant bioinformatics and molecular studies. As a member of the Caryophyllaceae family, carnation (*D. caryophyllus* L.) is one of the most widely used flowers in the world (Rhoads and Au, 2015; Taghizadeh and Khadivi, 2023). More than 300 *Dianthus* species were produced whereas many of their genomes remain to be sequenced. In the present study, we provide a chromosome-scale and haplotype-resolved genome assembly for *D. caryophyllus* genome of ‘Aili’, which is excellent cut flower with important ornamental and economic value.

Here, we generated the *D. caryophyllus* genome ‘Aili’ at the chromosome-level. Since the carnation is highly heterozygous, which was a great challenge for genome assembly, both homologous chromosomes were assembled. The haplotype-resolved assembled genome contains a total of 36.9 Gb PacBio HiFi reads. The genome sizes were 582.39 Mb for hap1 and 578.49 Mb for hap2, respectively. PacBio long reads overcome many limitations of genome assembly using previous sequencing technologies, for example, increased coverage resulted in great improvement of the accuracy (Sun et al., 2022). N50 is frequently used to assess genome assembly quality (Jauhal and Newcomb, 2021). The generated hap1 included 245 contigs with a N50 length of 19.84 Mb, hap2 included 189 contigs with a N50 length of 25.17 Mb. The N50 values were improved comparing that of the previously released carnation genome of ‘Scarlet Queen’ (Zhang et al., 2022). High N50 values of our genome indicated high assembly contiguity. The bioinformatic tool BUSCO is one main method for evaluating genome assembly. Compared with the former carnation genome assembly of ‘Scarlet Queen’, the BUSCO values of the genome assembly of ‘Aili’ were also improved. High BUSCO completeness rates (97.50% for hap1 and 97.40% for hap2) confirmed the excellent quality of the genome assembly.

Repetitive sequences are the driven force in the species evolution as they participate in numerous processes such as chromosome recombination and arrangement (Britten, 2010). In this study, we compared the repetitive sequence differences between ‘Aili’ and the previously published ‘Scarlet Queen’ and ‘Francesco’ in *D. caryophyllus*. The total number of the repetitive sequences of ‘Aili’ was basically consistent with that from carnation genome of ‘Scarlet Queen’ (Zhang et al., 2022). However, the previously reported ‘Francesco’ genome had much less repetitive sequences in total (Yagi et al., 2013). The differences on repetitive sequences may impact on phenotypic variation by modelling the regulatory patterns of genes (Knight, 2004). The three varieties ‘Aili’, ‘Scarlet Queen’ and ‘Francesco’ differed in flower colors. The petal color of single-flowered ‘Aili’ is in red with yellow gradient. Double-flowered ‘Scarlet Queen’ is basically in red with a little white color at the petal edges. Double-flowered ‘Francesco’ is in red. Our GO annotation on the unique gene families of the three carnations demonstrated that ‘Aili’-specific genes were mainly enriched cytochrome complex assembly, whereas ‘Francesco’-specific genes and ‘Scarlet Queen’-specific genes were mainly enriched in RNA-directed DNA polymerase activity and nucleic acid metabolic process, respectively. As cytochromes play important roles in biosynthesis of major floral pigments (Tanaka and Brugliera, 2013), the enriched cytochrome complex assembly in ‘Aili’-specific genes may explain the color difference of three varieties.

In summary, we provide a chromosome-scale and haplotype-resolved genome assembly of carnation (*D. caryophyllus*) using PacBio sequencing and Hi-C technology. This high-quality genome will provide a valuable genome resource for the domestication and evolutionary studies of *D. caryophyllus*. Moreover, this gives us an opportunity to identify genes in the carnation genome and further provides a basis for molecular biology studies which contribute to economic production.

## Data availability statement

All original contributions presented in the study are publicly available. The genome assembly and annotation data presented in the study were deposited on FigShare at the link: https://doi.org/10.6084/m9.figshare.23808174.v3 and on the China National GeneBank (https://db.cngb.org/) under project number CNP0004649.

## Author contributions

ZW, XNZ and YY: conceptualization. HJ, XNZ, LL, DG, XHZ, JL, DP and YY: formal analysis. HJ and YY: writing—original draft. ZW, HJ, XNZ and YY: writing—review and editing. All authors have read and agreed to the published version of the manuscript.
