# Supplementary material for: A chromosome-scale and haplotype-resolved genome assembly of carnation (Dianthus caryophyllus) based on high-fidelity sequencing
Source: Front Plant Sci. 2023 Aug 4;14:1230836. doi: 10.3389/fpls.2023.1230836 (PMC10437072; doi:10.3389/fpls.2023.1230836)
Supplement: Supplementary file 1 [file DataSheet_1.docx]

Supplementary Material

A chromosome-scale and haplotype-resolved genome assembly of carnation (*Dianthus caryophyllus*) based on high-fidelity sequencing

Heling Jiang^1,2†^, Xiaoni Zhang^1†^, Luhong Leng^2^, Desheng Gong^2^, Xiaohui Zhang^2^, Junyang Liu^2^, Dan Peng^1^, Zhiqiang Wu^1,2^*, Yingxue Yang^1,2^*

^1^Kunpeng Institute of Modern Agriculture at Foshan, China.

^2^Agricultural Genomics Institute at Shenzhen, Chinese Academy of Agricultural Sciences, China

†These authors contributed equally to this work.

*** Correspondence:**

Yingxue Yang: yangyingxue@caas.cn

Zhiqiang Wu: wuzhiqiang@caas.cn

# Supplementary Figures


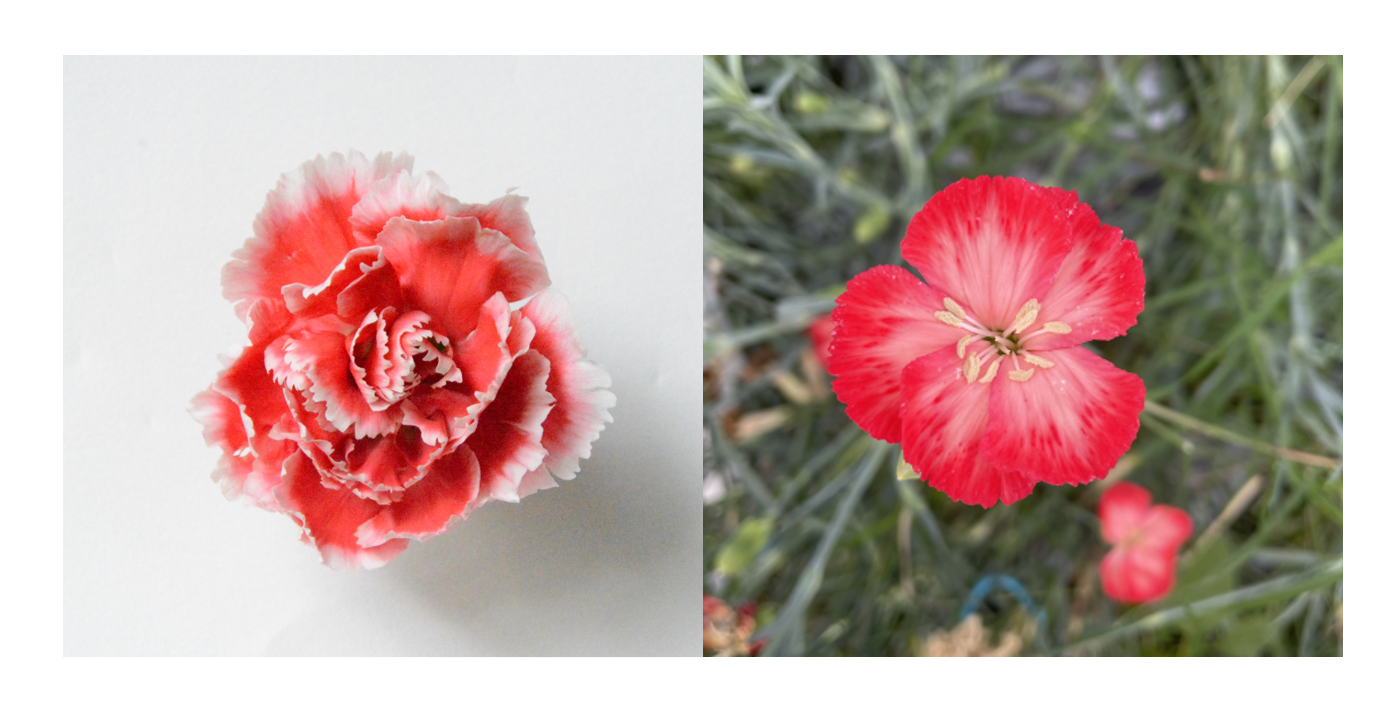


**Supplementary Figure 1.** Morphological photos of flowers of *D. caryophyllus* ‘Scarlet Queen’ (left) and *D. caryophyllus* ‘Aili’ (right).


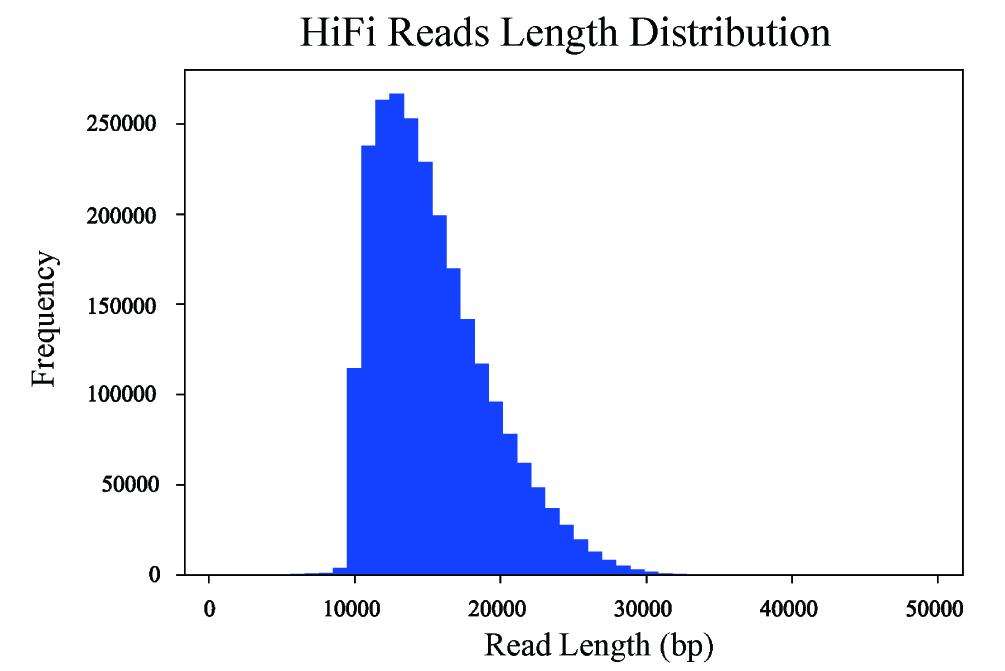


**Supplementary Figure 2.** Hifi reads length distribution.


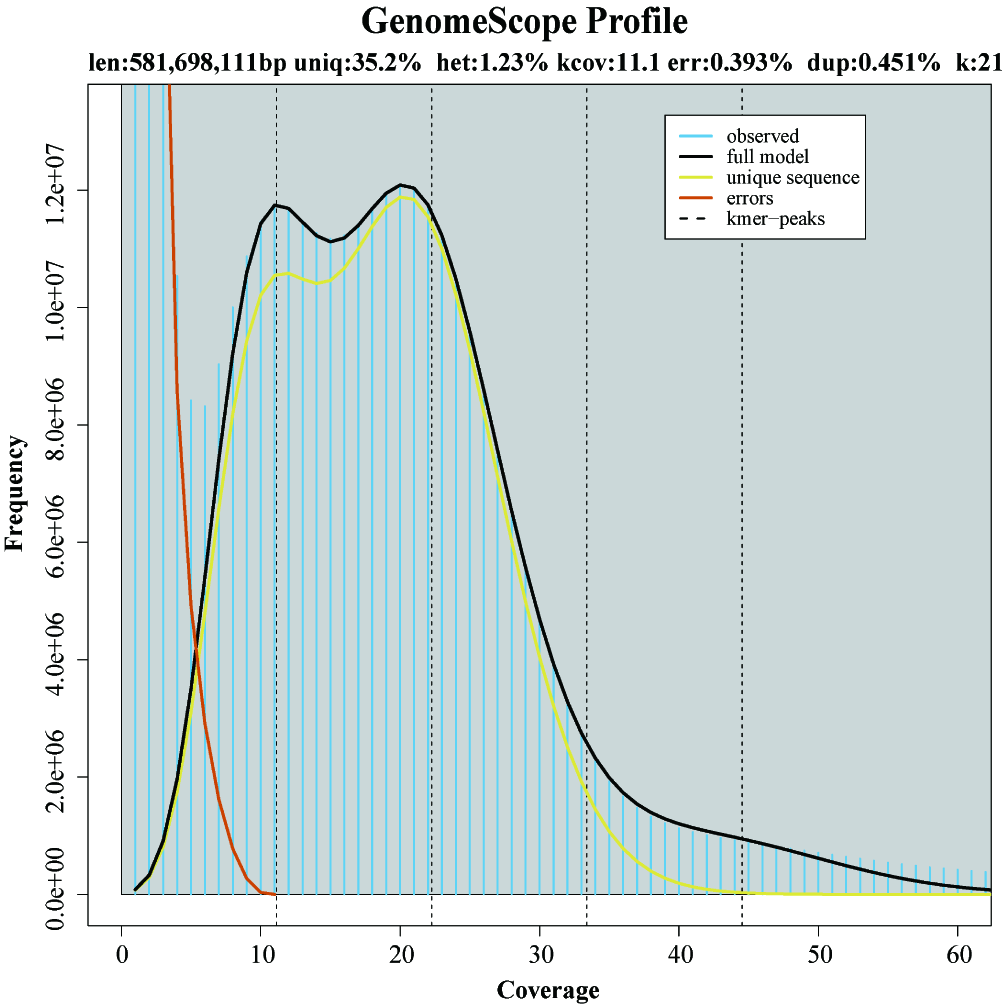


**Supplementary Figure 3.** Estimation of genome size, heterozygosity, and repetitive sequences using *k*-mer analysis.


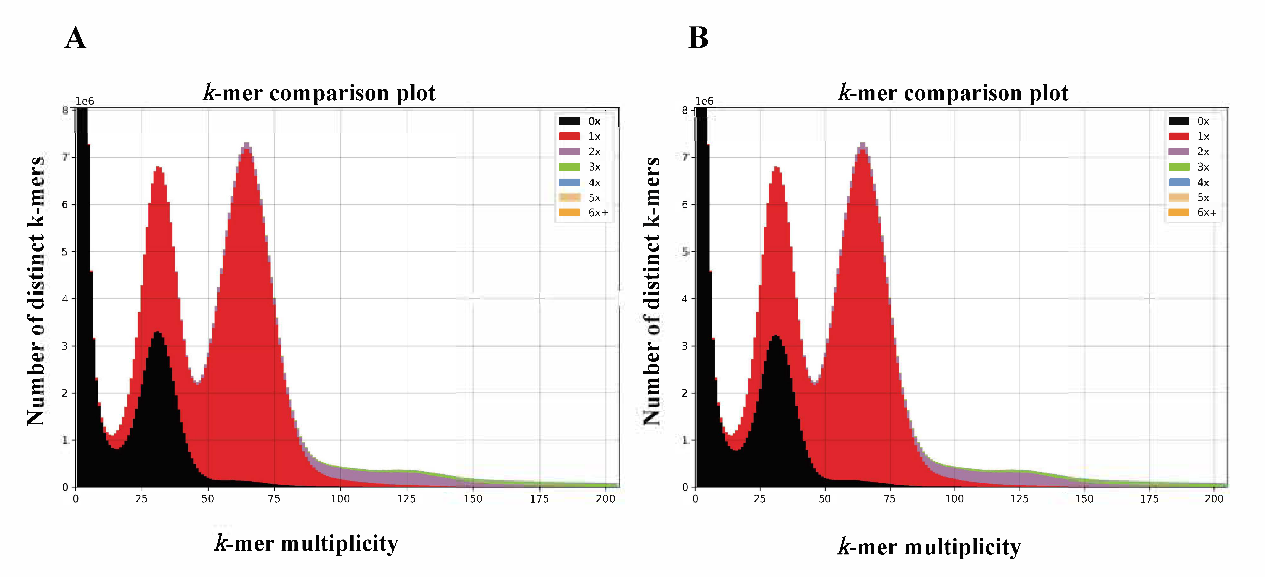


**Supplementary Figure 4.** *k*-mers level comparative analysis of two haplotype results of hifiasm assembly and original reads. **(A)** The result of haplotype 1 **(B)** The result of haplotype 2.


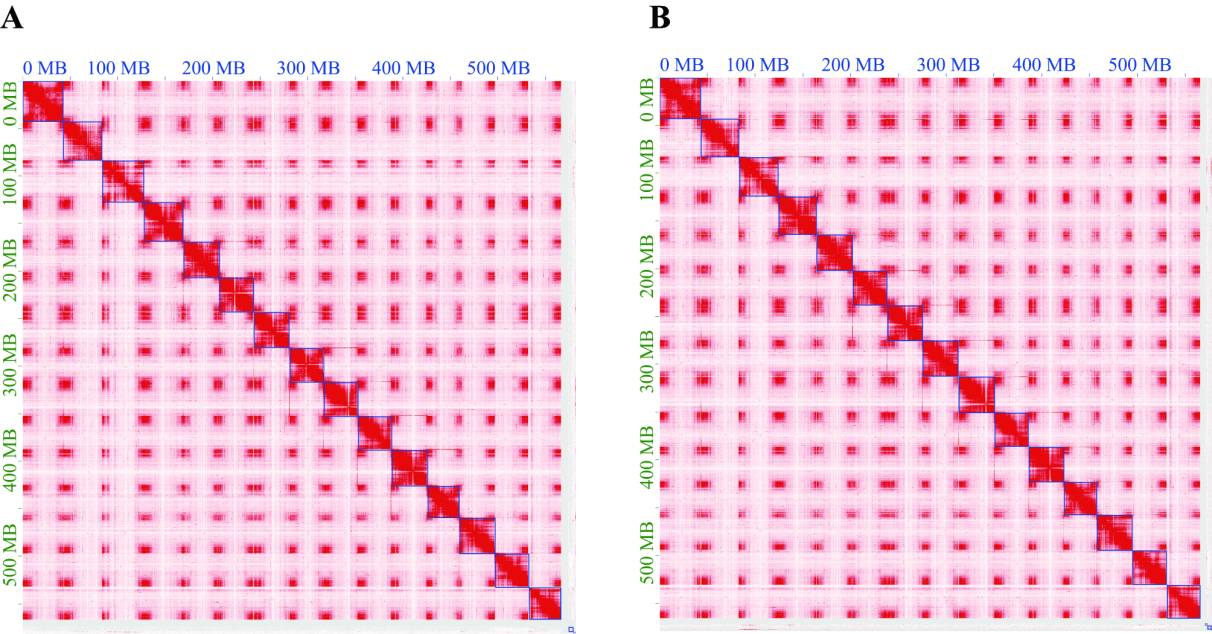


**Supplementary Figure 5.** Hi-C contact maps to the two final haplotype assemblies. **(A)** The result of haplotype 1 **(B)** The result of haplotype 2.


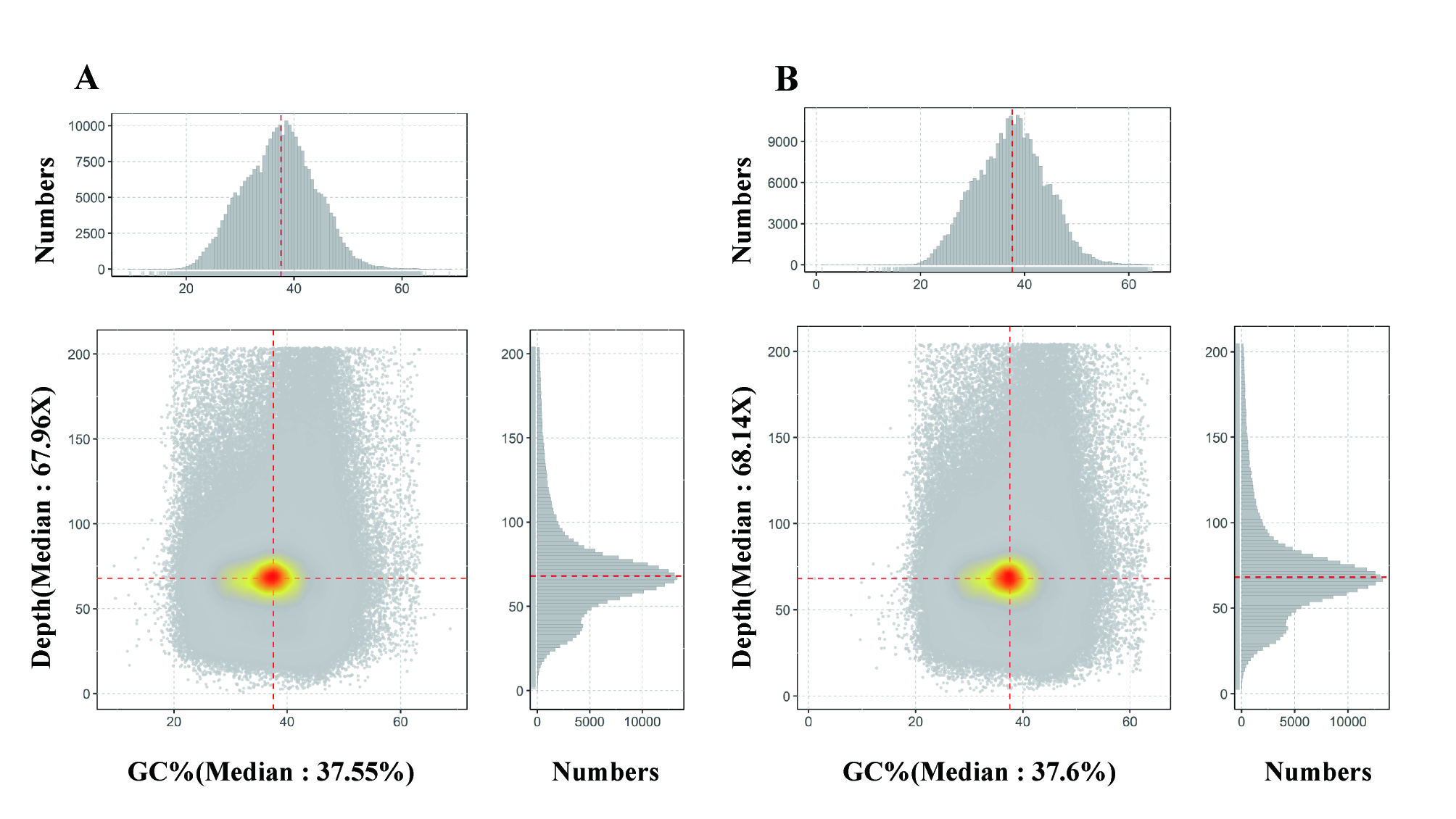


**Supplementary Figure 6.** Sequence depth and GC content for the carnation genome assemblies. **(A)** The result of haplotype 1 **(B)** The result of haplotype 2.

**
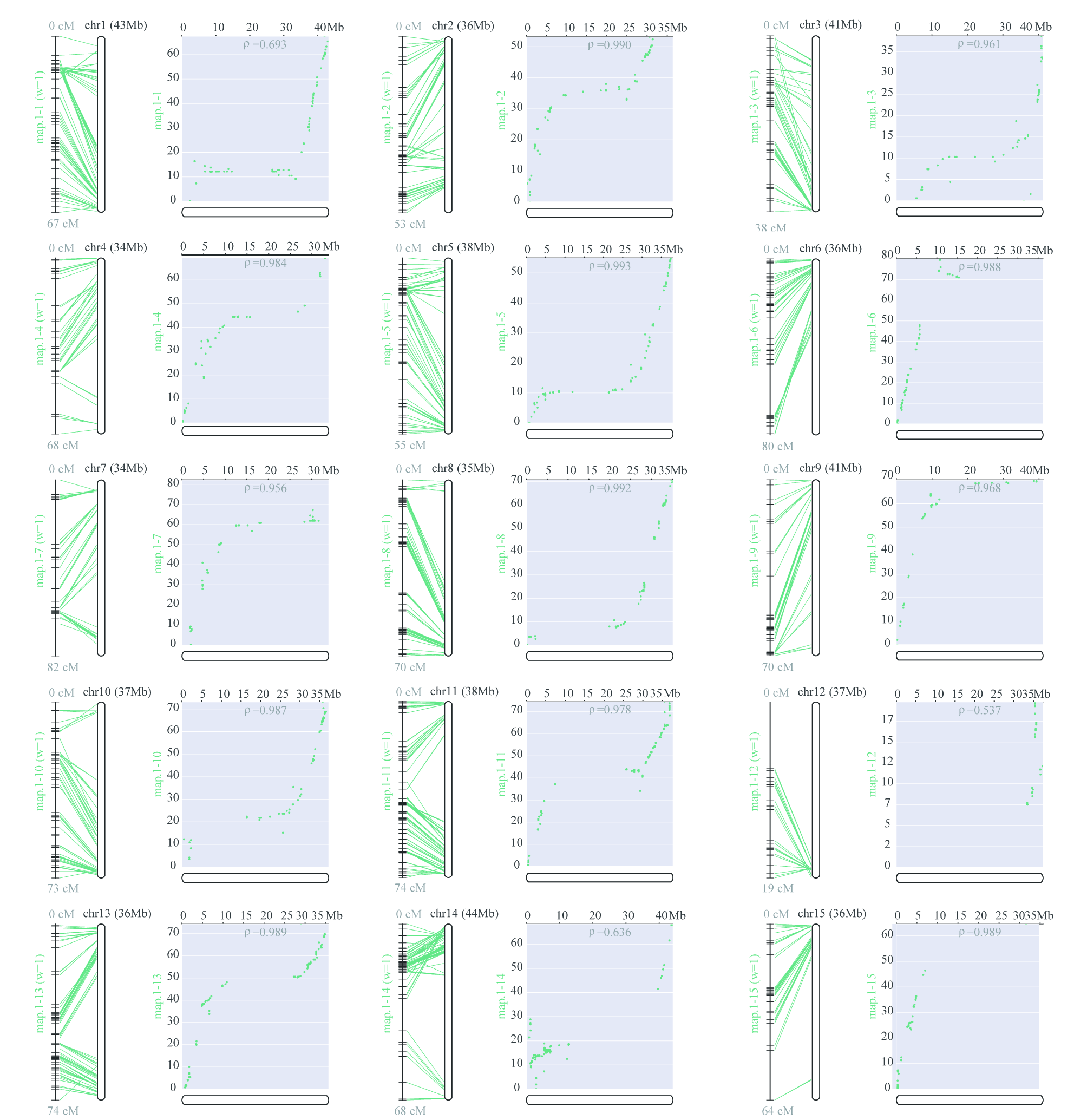
**

**Supplementary Figure 7.** Collinearity between the *D. caryophyllus* ‘72L’ genetic map and *D. caryophyllus* ‘Aili’ haplotype 1 pseudomolecules.

**
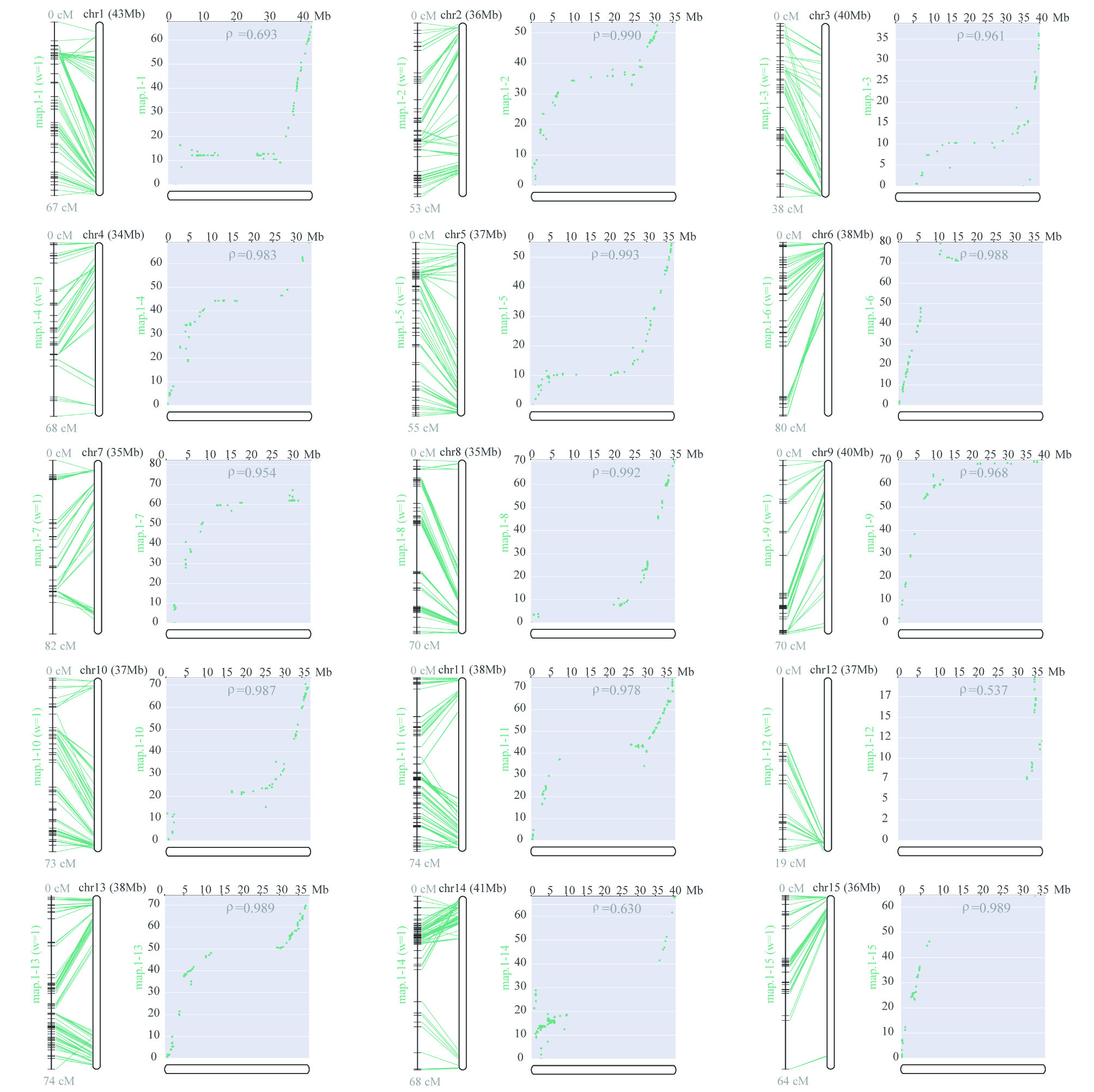
**

**Supplementary Figure 8.** Collinearity between the *D. caryophyllus* ‘72L’ genetic map and *D. caryophyllus* ‘Aili’ haplotype 2 pseudomolecules.

**
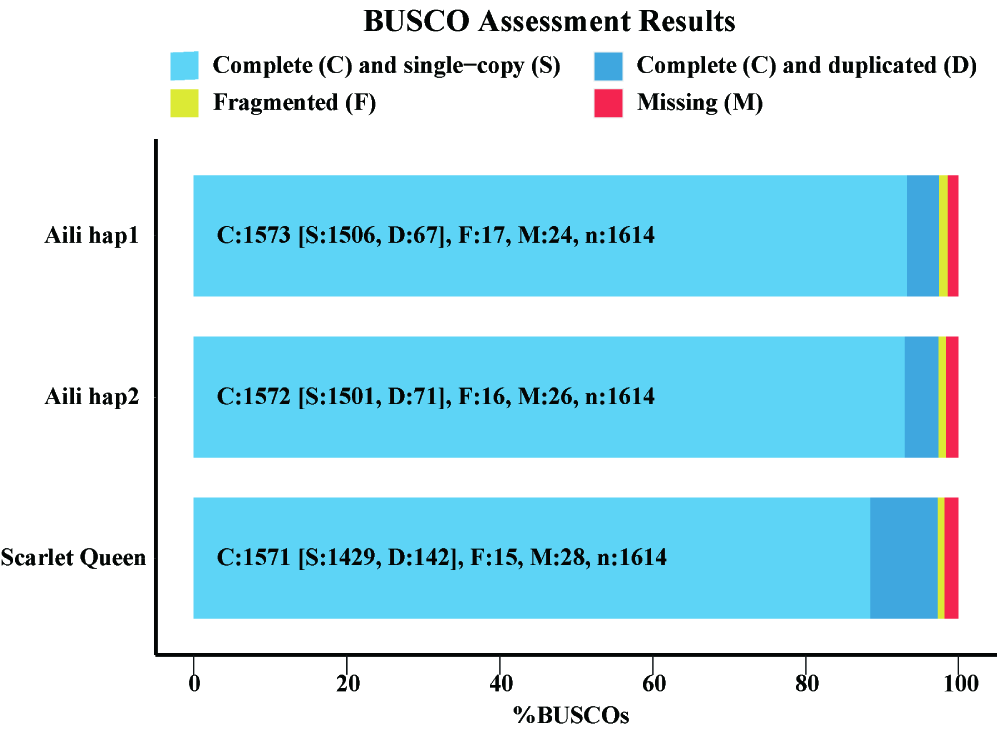
**

**Supplementary Figure 9.** Comparison of Busco results between the genomes of *D. caryophyllus* ‘Scarlet Queen’ and *D. caryophyllus* ‘Aili’.


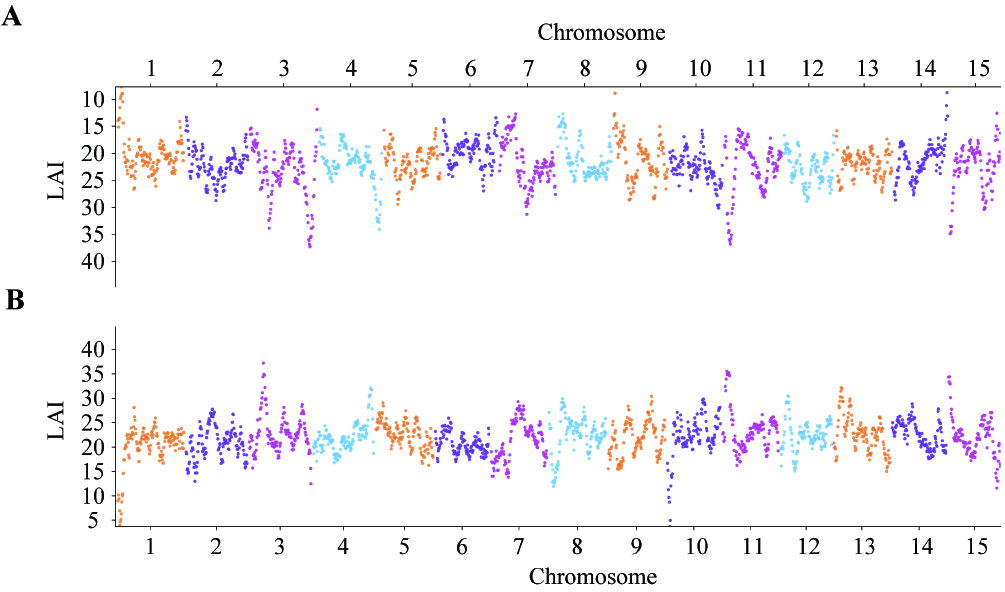


**Supplementary Figure 10.** The statistical results of LAI of *D. caryophyllus*. **(A)** The result of haplotype 1 **(B)** The result of haplotype 2.

**
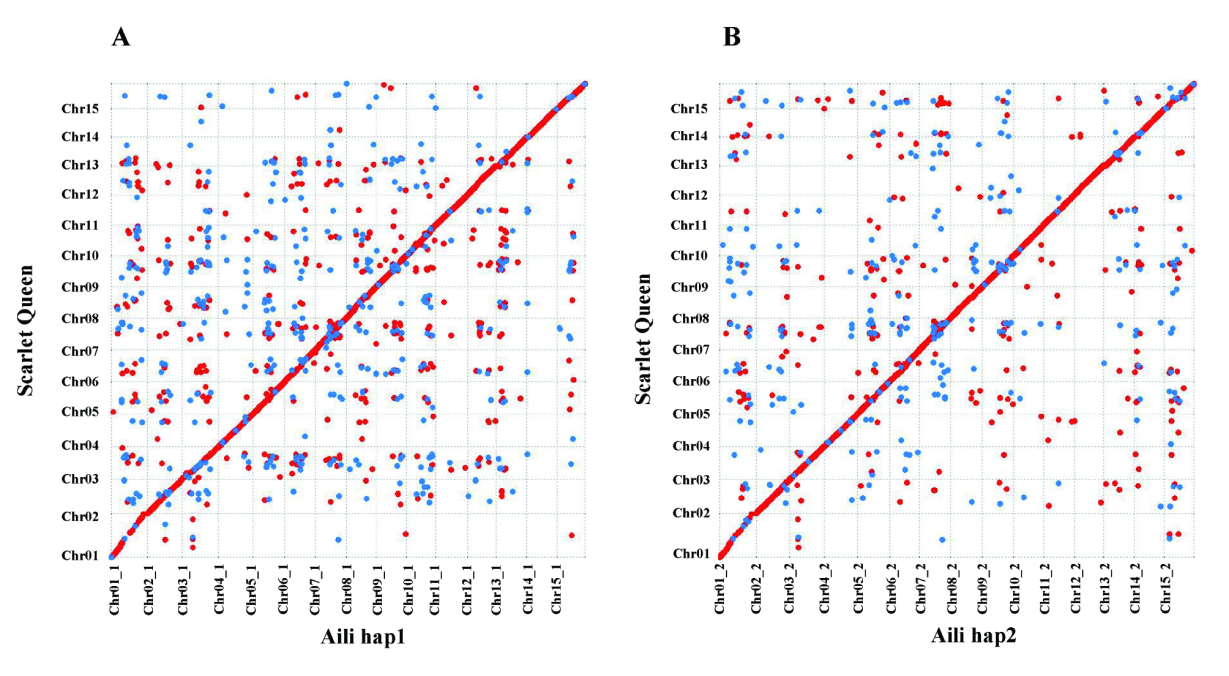
**

**Supplementary Figure 11.** Collinear dot map between the genomes of *D. caryophyllus* ‘Scarlet Queen’ and *D. caryophyllus* ‘Aili’.


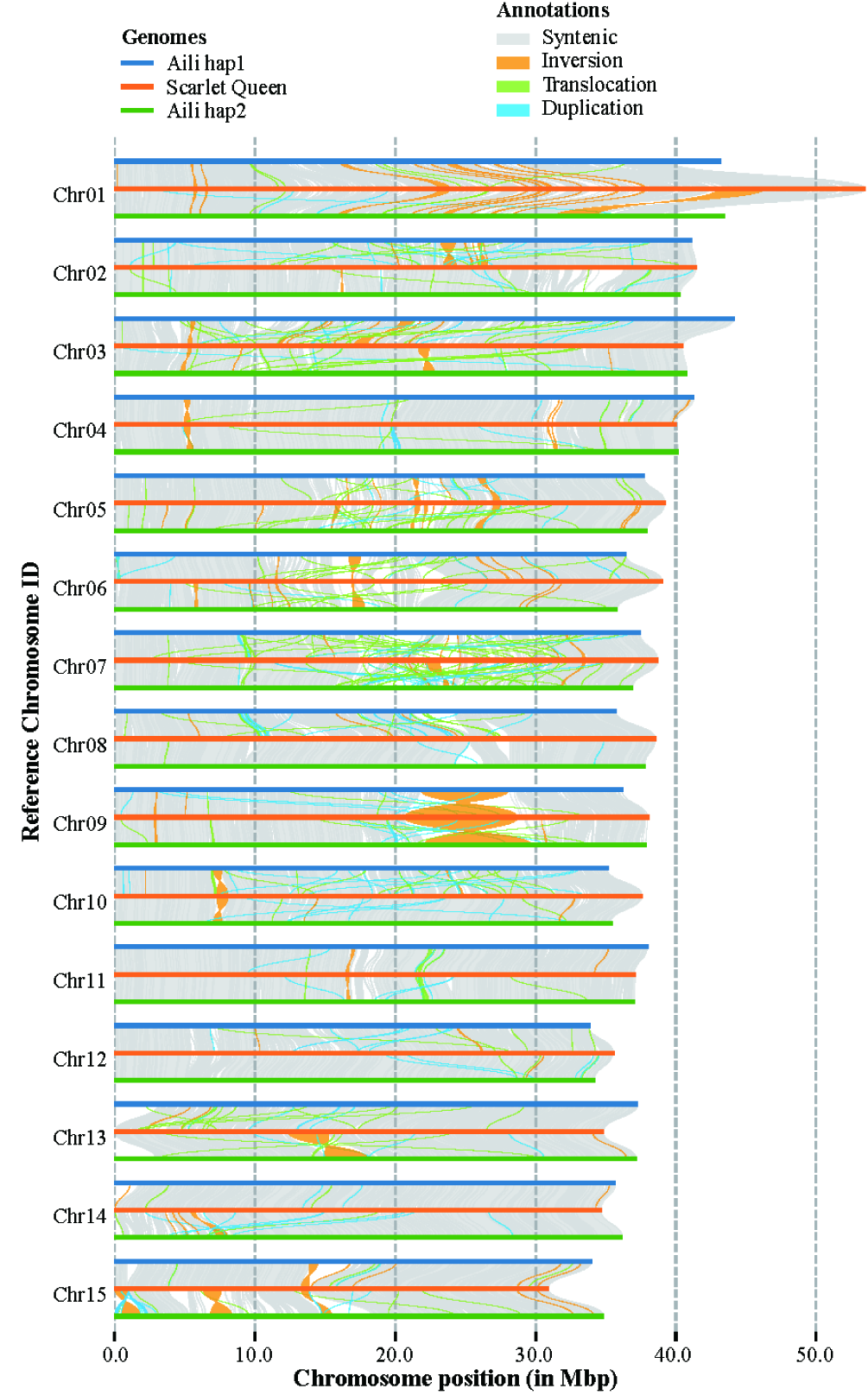


**Supplementary Figure 12.** Collinearity relationships between the genomes of *D. caryophyllus* ‘Scarlet Queen’ and *D. caryophyllus* ‘Aili’.


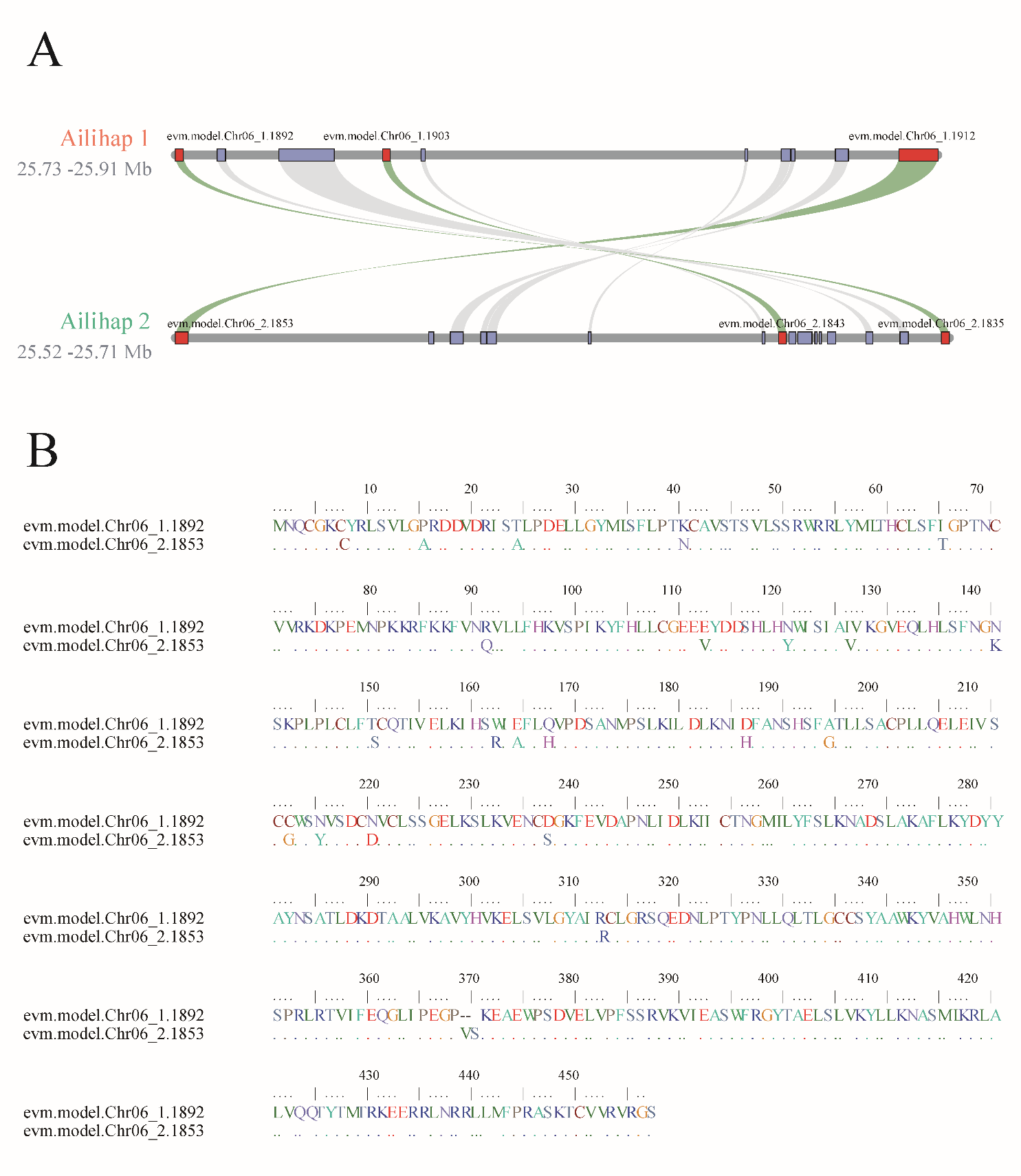


**Supplementary Figure 13.** Syntenic analysis in ‘Aili’. **(A)** Syntenic region illustrating structural variations between hap1 and hap2. **(B)** *FBL13-like* gene variation between hap1 and hap2 on the syntenic regions.


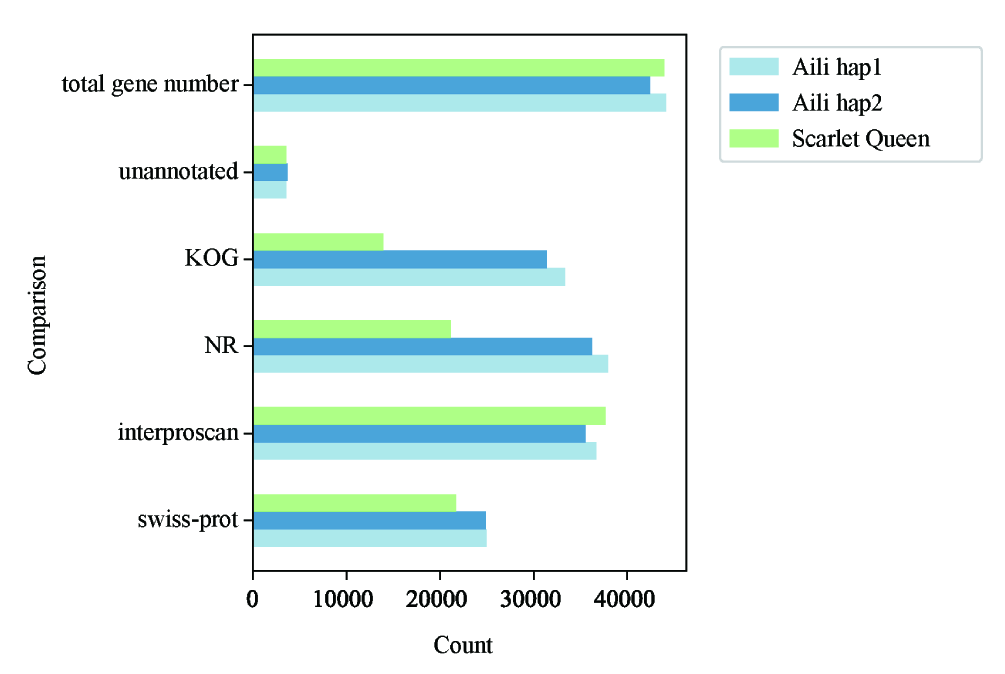


**Supplementary Figure 14.** The statistical results of gene function annotation of *D. caryophyllus*.


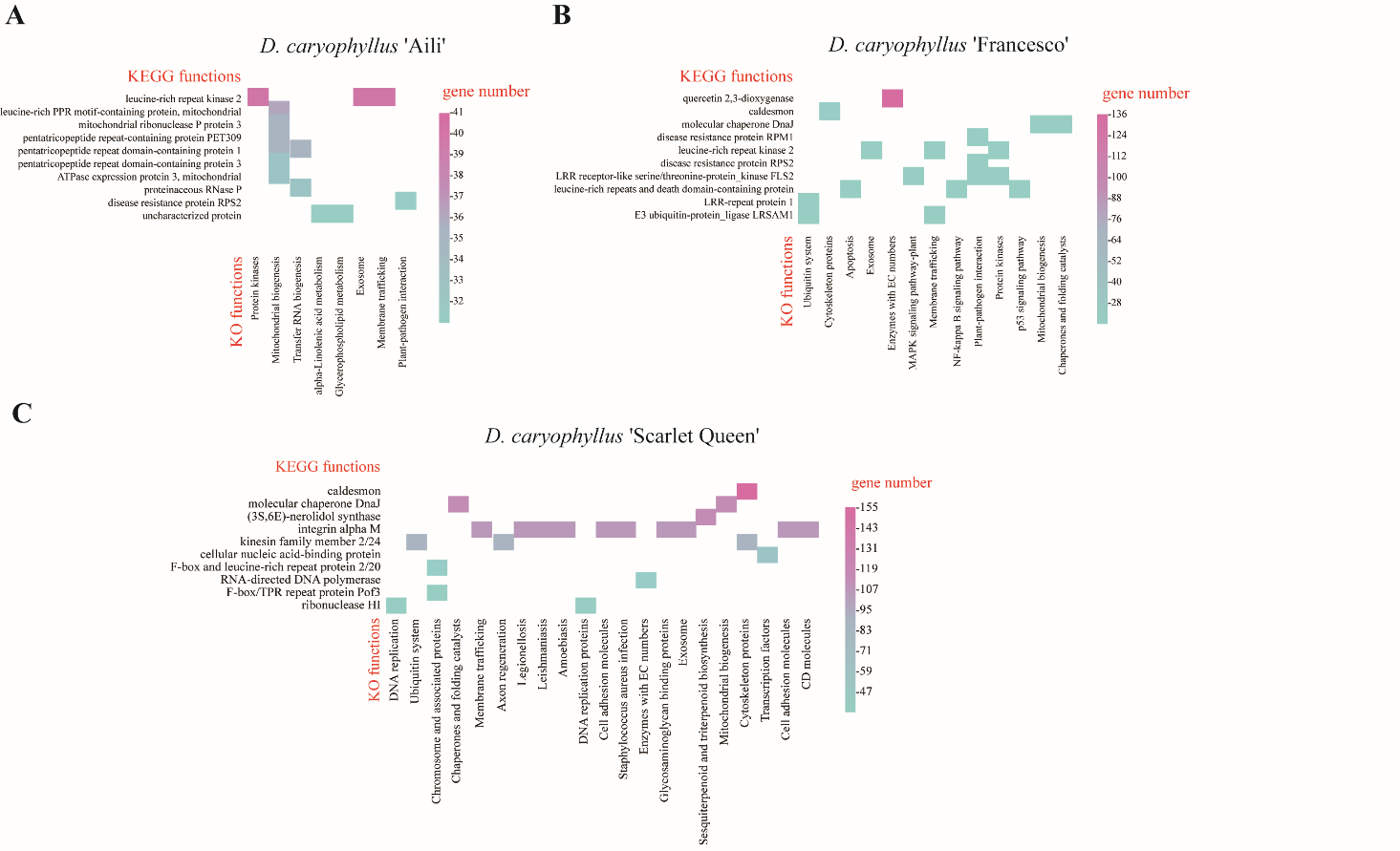


**Supplementary Figure 15.** KEGG analysis of the unique gene family among the three carnation genomes. **(A)** *D. caryophyllus* ‘Aili’ **(B)** *D. caryophyllus* ‘Francesco’ **(C)** *D. caryophyllus* ‘Scarlet Queen’.


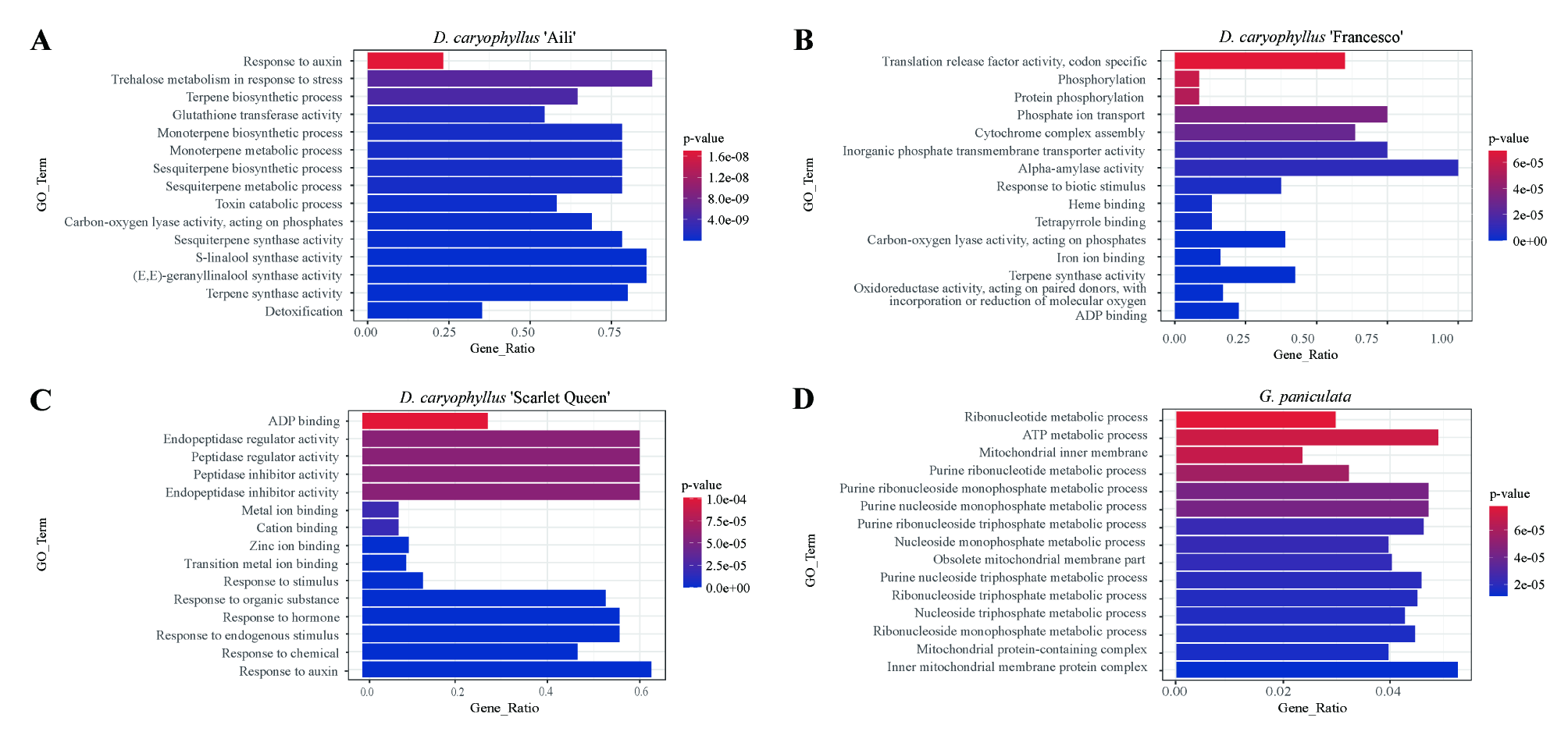


**Supplementary Figure 16.** GO enrichment analysis of the expanded gene family under the order Dianthus. **(A)** *D. caryophyllus* ‘Aili’ **(B)** *D. caryophyllus* ‘Francesco’ **(C)** *D. caryophyllus* ‘Scarlet Queen’ **(D)** *G. paniculate*.


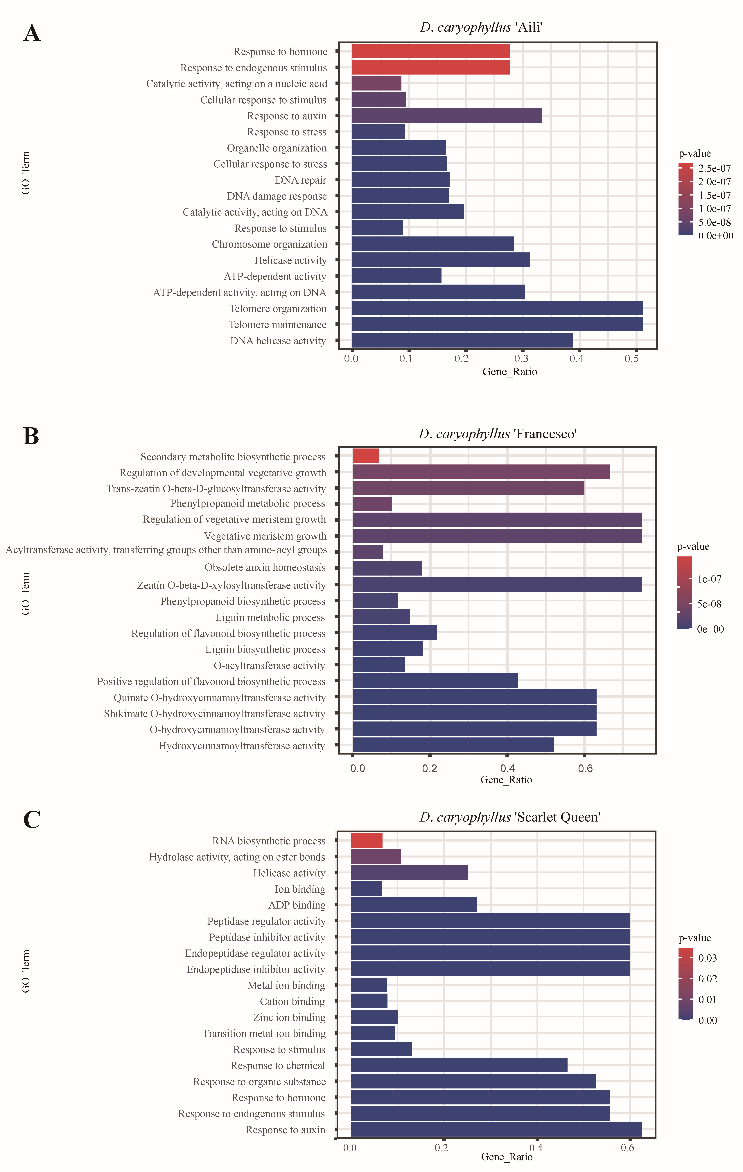


**Supplementary Figure 17.** GO enrichment analysis of the contracted gene family under the order Dianthus. **(A)** *D. caryophyllus* ‘Aili’ **(B)** *D. caryophyllus* ‘Francesco’ **(C)** *D. caryophyllus* ‘Scarlet Queen’.
